# Telomere length and oxidative stress in small-cell lung cancer: commentary on prognostic value

**DOI:** 10.11613/BM.2026.010401

**Published:** 2025-12-15

**Authors:** Seshadri Reddy Varikasuvu

**Affiliations:** Department of Biochemistry, All India Institute of Medical Sciences, Deoghar, India

**Keywords:** chemotherapy, oxidative stress, paraoxonase-1, small-cell lung cancer, telomere length

## Abstract

This commentary discusses the prognostic relevance of leukocyte telomere length and paraoxonase-1 activity in small-cell lung cancer (SCLC) patients undergoing chemotherapy. It emphasizes the importance of integrating telomere biology and oxidative stress assessment in prognostic modeling. The discussion also considers the modifying effects of lifestyle, treatment regimens, and genetic background, advocating for research that combines clinical, biochemical, and molecular data to enhance prognostication in SCLC.

## Graphical abstract

**Figure:**
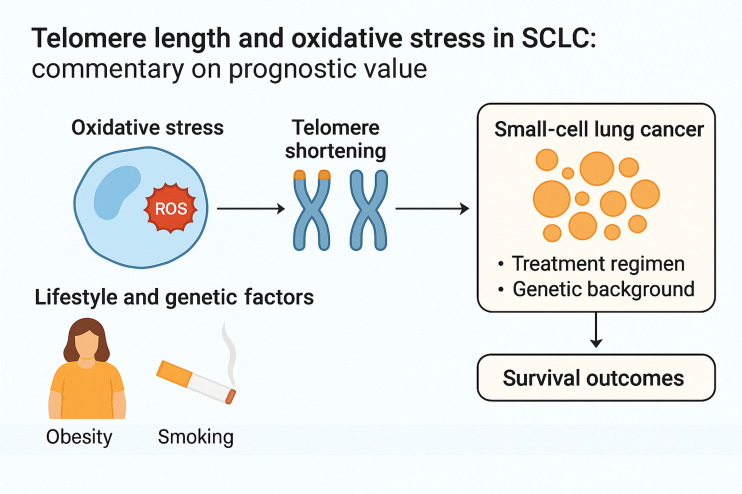

## Highlights

• The letter contextualizes the prognostic role of leukocyte telomere length and paraoxonase-1 activity in small-cell lung cancer (SCLC), in response to recent findings by Guzonjić *et al.*

• It highlights the influence of oxidative stress, lifestyle factors (obesity and smoking), treatment regimens, and genetic background on telomere dynamics and clinical outcomes

• The commentary underscores the importance of integrating telomere biology with clinical variables and molecular markers for improved prognostication in SCLC

• It encourages future research incorporating platinum sensitivity, genetic polymorphisms, and larger cohorts to enhance clinical applicability

## Dear Editor,

I read with great interest the recent article by Guzonjić *et al*., which examined leukocyte telomere length (LTL) and paraoxonase-1 (PON1) activity in patients with small-cell lung cancer (SCLC), providing insights into their prognostic significance using Kaplan–Meier and Cox regression models (*1*). The study makes an important contribution to understanding how telomere biology and oxidative stress may influence cancer outcomes. The inclusion of LTL and redox markers across three treatment time points offers valuable information on chemotherapy-induced changes in systemic oxidative stress and survival. Obesity and smoking are recognized sources of oxidative stress and telomere attrition. Adjusting for these factors, as performed by Guzonjić *et al*., strengthens the validity of the reported associations between LTL, PON1, and survival (*1*). The reported hazard ratios (HR = 1.747 for LTL and HR = 1.710 for PON1) were directionally consistent with prior evidence, underscoring the importance of considering lifestyle-related confounders in biomarker-based prognostication models.

Emerging data suggest that clinical outcomes in SCLC are influenced not only by baseline redox status and telomere dynamics but also by treatment regimens and genetic background. For instance, platinum-based chemotherapy has been shown to induce oxidative DNA damage and affect telomere dynamics in experimental models. Chemotherapy-induced oxidative damage and genetic background may further modulate survival in SCLC. Platinum-based regimens can affect telomere dynamics, while polymorphisms in DNA repair or telomere-maintenance genes influence treatment response (*2*). Although the study by Guzonjić *et al*. do not stratify outcomes by platinum sensitivity or DNA damage response markers, such analyses would enhance future investigations.

Findings from meta-analyses further contextualize the present results. Studies aggregating telomere length data across multiple cancer types support a consistent association between shorter LTL and poor prognosis, although variability remains due to methodological and population differences (*3*, *4*). Similarly, reduced PON1 activity has been linked to poor outcomes in SCLC and may interact with telomere attrition in a synergistic fashion (*3*).

Evidence from Mendelian randomization studies reveals a complex interplay between genetically determined telomere length and cancer risk. While shorter telomeres are generally associated with genomic instability and increased susceptibility to cancer, longer telomeres in certain contexts may facilitate unchecked proliferation and tumor progression (*5*). These nuances emphasize that LTL may function as both a biomarker and potential mediator in SCLC biology, requiring careful interpretation.

The hazard ratios (HRs) were derived from multivariate Cox models adjusted for age, body mass index, smoking status, disease stage, performance status, chemotherapy cycle, and baseline redox and telomere values. Although not statistically significant, the HRs were directionally consistent with prior evidence, underscoring the robustness of the analysis (*1*). Notably, the survival curves for both LTL and PON1 began to diverge after the median survival time, suggesting a potential late effect that might be clarified with larger cohorts or longer follow-up.

Overall, the study by Guzonjić *et al.* provides meaningful insight into telomere-redox dynamics in SCLC. Future research integrating telomere biology, oxidative stress markers, and genetic modifiers could further enhance prognostic clinical utility and therapeutic strategies.

## Data Availability

Data sharing not applicable to this article as no datasets were generated or analysed during the current study.
